# Coronavirus biopolitics: the paradox of France’s Foucauldian heritage

**DOI:** 10.1007/s40656-020-00359-2

**Published:** 2021-01-11

**Authors:** Mathieu Arminjon, Régis Marion-Veyron

**Affiliations:** 1 School of Health Sciences (HESAV), University of Applied Sciences and Arts Western Switzerland (HES-SO), Avenue de Beaumont 21, 1011 Lausanne, Switzerland; 2 Center for Primary Care and Public Health (Unisanté), University of Lausanne, Rue du Bugnon 44, 1011 Lausanne, Switzerland

**Keywords:** COVID-19, France, Foucault, Biopolitics, Epidemiological surveillance, Social inequalities in health

## Abstract

In this short paper we analyse some paradoxical aspects of France’s Foucauldian heritage: (1) while several French scholars claim the COVID-19 pandemic is a perfect example of what Foucault called *biopolitics*, popular reaction instead suggests a *biopolitical failure* on the part of the government; (2) One of these failures concerns the government’s inability to produce reliable biostatistical data, especially regarding health inequalities in relation to COVID-19. We interrogate whether Foucaldianism contributed, in the past as well today, towards a certain myopia in France regarding biostatistics and its relation to social inequalities in health. One might ask whether this very data could provide an appropriate response to the Foucauldian question: What kind of governance of life is the pandemic revealing to us?

French scholars^1^ have often stated that the COVID-19 pandemic demonstrates “coronavirus biopolitics” (Zarka 2020, our translation). Foucauldian “biopolitics” is the critique of a historically contextualised mode of governance that emerged alongside the liberal state in the nineteenth century. In biopolitical governance, citizens are not subjects of law, but a *biological* population to be controlled by means of epidemiological (biostatistical) surveillance (Foucault 1997[1976]). Seemingly, then, we are experiencing a “Foucauldian moment” (Cot 2020). The more coronavirus spreads, the more population surveillance becomes apparent.

Yet, widespread reaction against the political management of the crisis in France conflicts with this reading: people accuse the government of failing to manage the pandemic and are calling for the release of socioeconomic statistics to fully assess the extent of this *biopolitical failure*. But the biostatistical data linking COVID-19 mortality to socio-economic status is missing (Naiditch and Lombrail 2020). Notably, unlike elsewhere, it was not *until* the crisis that some epidemiologists lamented the lack of research on social determinants of health in France. Ironically, Foucault’s influence is never discussed; some historical evidence suggests that critiques of biopolitics might have impeded research concerning social determinants of health in France.

This is the French paradox we intend to interrogate^2^ here by means of a brief exploration of two hypotheses: (1) the pandemic reveals less a “coronavirus biopolitics” than a biopolitical failure and, (2) a deep-seated Foucauldian suspicion of biostatistics perhaps contributed—and still contributes—to a lack of data in France regarding social vulnerability to disease. Ultimately, this prompts us to reflect on the paradoxical implications of referring to Foucault in the context of a pandemic, especially regarding how to measure social inequalities in health.

The French government was particularly criticized for its unpreparedness and slow response to the crisis (Deléan 2020). Journalists and experts have conducted what we could call “comparative biopolitics”, as the responses of South Korea, Taiwan, Portugal, and Germany were praised for having lower death rates than France. French citizens have even been encouraged to sue the government for endangering people’s lives more than in other nations.^3^

Undoubtedly, surveillance and control are potential threats to public freedom. But to the people, this crisis highlights France’s inadequate health infrastructure, debilitated by several decades of austerity politics. Bruno Latour recently discussed how the crisis questions the current governance of life; some see COVID-19 as an opportunity to imagine a more egalitarian world, while “globalizers” see it as a dream opportunity “to get rid of the rest of the welfare state, the safety net for the poorest (…) and, more cynically, to get rid of all those supernumerary people who encumber the planet” (Latour 2020, our translation).

In the 1970s, Foucault condemned the risks of a potentially authoritarian State armed with epidemiological technologies like biostatistics. But in times of pandemic crisis, people have come to value the benefits of such technologies. Latour’s words echo the unprecedented popular accusation of the government’s biopolitical failure. In the same vein, public opinion seems to express that the notion of epidemiological surveillance—understood as a restraint on public freedom—is outweighed by its potential for social protection and good public health policy. People have internalized what social epidemiologists have been claiming for several decades: yes, biostatistics can be used as a technology of social control, but also as a tool for social empowerment.

One of social epidemiology’s foremost contributions has been to use statistics to demonstrate that “the higher the social position, the better the health” (Marmot 2006, p. 1304). This pandemic is no exception: one of France’s poorest neighbourhoods, the Seine Saint-Denis, has one of the highest mortality rates (Mariette and Pitty 2020). Some scholars have thus denounced the lack of public data available for analysing COVID-19 death rates in correlation with socio-professional and ethno-racial status.^4^

History sheds some light on France’s research void concerning social determinants of health. In 1980, the UK’s Black Report (Black 1980), based on census statistics, showed that general mortality had fallen between the 1950′s and 1970′s, but that the mortality gap between the lowest and highest social classes had risen. The report’s publication became the subject of global political debate in many other countries and led to much research. At the same time, in France, one of the few articles on the subject (written by an epidemiologist) concluded by criticizing social epidemiology, explicitly referencing Foucault: “At the crossroads of the life sciences and the human sciences, epidemiology [of social factors] now seems to be one of the most complete forms of the influence exerted since the end of the nineteenth century on the social sciences by a biological model based on the notion of norm” (Goldberg 1982, p. 99, our translation). Did a Foucauldian mindset slow the development of French social epidemiology and render any collection of socioeconomic biostatistics suspect due to the risk of social control?

That said, let’s return to the two aspects of France’s Foucauldian paradox:

1. In the context of a weakening welfare state, the crisis seems not to expose the risk of social control but, rather, the biopolitical failures of the state. This raises questions *Foucauldian orthodoxy* cannot address, but which are Foucauldian *par excellence*: in a time of pandemic, what kind of governance is revealed by the biopolitical failure of the State? Is globalization potentially carrying us, as Latour states, into a biopolitics where life is no longer a political object, or where the lives of those who cannot afford healthcare are worth less than those who can?
2. This crisis reveals an absence of epidemiological surveillance linked to the biopolitical failure of the state. Yet, we have also good reason to suggest that a predominance of critical Foucauldian thought has, in fact, considerably stunted a French culture of research on the social determinants of health. In a period of pandemic crisis, a Foucauldian denunciation of the risks of social control by means of biostatistics might still lead to a myopia amongst French researchers regarding social inequalities in health.

If so, it seems French researchers—more generally we, Foucault-fed scholars—should be more interrogative as to the paradoxical outcomes tied to claiming that the crisis reveals a biopolitics of coronavirus. In doing so, are we not indirectly prolonging a suspicion of epidemiological surveillance, when it is in fact biostatistical data that may help us fully understand (and potentially criticize) the type of governance that the pandemic is currently exposing?

## Footnotes

1 We should point out at the outset that both authors are French-Swiss, living and working in French speaking Switzerland. Our interest in the French situation stems as much from our origins as from shared academic traditions with France.

2 This note belongs to the Topical Collection “Seeing Clearly Through COVID-19: Current and Future Questions for the History and Philosophy of the Life Sciences”, edited by G. Boniolo and L. Onaga.

3 For instance, see https://www.coronavictimes.fr/

4 The French administration does not publish statistics relating to the socio-economic status of victims of COVID-19. A Foucauldian mistrust of biostatistics indirectly aligns itself with an “assimilationist” French model hostile to any ethno-racial categorization (Simon 1997). The lack of statistics precludes any drift toward stigmatization. But it also prohibits objectivity and thus the denunciation of possible inequalities related to racial discrimination. In the United States, the culture of ethno-racial categorization has made it possible to draw particular attention to the excess mortality due to COVID in the African American population. See for instance the figure published by the NYC Department of Health and Mental Hygiene in April 2020: https://www1.nyc.gov/assets/doh/downloads/pdf/imm/covid-19-deaths-race-ethnicity-04162020-1.pdf
